# A Single Molecule Investigation of the Photostability of Quantum Dots

**DOI:** 10.1371/journal.pone.0044355

**Published:** 2012-08-31

**Authors:** Eva Arnspang Christensen, Pasad Kulatunga, B. Christoffer Lagerholm

**Affiliations:** 1 Department of Physics and Chemistry and MEMPHYS – Center for Biomembrane Physics, University of Southern Denmark, Odense M, Denmark; 2 Department of Physics, Hobart and William Smith Colleges, Geneva, New York, United States of America; Clarkson University, United States of America

## Abstract

Quantum dots (QDs) are very attractive probes for multi-color fluorescence imaging in biological applications because of their immense brightness and reported extended photostability. We report here however that single QDs, suitable for biological applications, that are subject to continuous blue excitation from a conventional 100 W mercury arc lamp will undergo a continuous blue-switching of the emission wavelength eventually reaching a permanent dark, photobleached state. We further show that β-mercaptoethanol has a dual stabilizing effect on the fluorescence emission of QDs: 1) by increasing the frequency of time that a QD is in its fluorescent state, and 2) by decreasing the photobleaching rate. The observed QD color spectral switching is especially detrimental for multi-color single molecule applications, as we regularly observe spectral blue-shifts of 50 nm, or more even after only ten seconds of illumination. However, of significant importance for biological applications, we find that even small, biologically compatible, concentrations (25 µM) of β-mercaptoethanol has a significant stabilizing effect on the emission color of QDs, but that greater amounts are required to completely abolish the spectral blue shifting or to minimize the emission intermittency of QDs.

## Introduction

Semiconductor quantum dots (QDs) have received much interest and use in a wide range of *in vitro* and *in vivo* biological applications including at the single molecule level for single particle tracking (SPT) [1], [2], [3], [4]. In contrast to conventional fluorescent dyes and proteins, QDs are much brighter and more photostable. QDs are also better suitable in multiplexing applications because QDs have narrow fluorescence emission spectra, with FWHM typically ranging from 20–50 nm. Furthermore, the fluorescence emission spectra of QDs lack the asymmetric slow decaying spectral tails that makes multiplexing with conventional dyes and proteins challenging.

QDs are also available in a wide range of emission colors, from the visible to the near-IR, where all QDs have overlapping excitation spectra such that they can be excited at any wavelength less than the emission peak of the bluest QDs in a particular multiplexing experiment. The multiplexing capability of QDs for multi-color SPT has already been demonstrated for two colors with QDs emitting at 585 and 655 nm [1], [5].

A major disadvantage of QDs, however, is the commonly observed emission intermittency, with in particular commercially available hybrid CdSe/CdTe QDs spending a majority of their time in a non-fluorescent dark state [6], [7], [8], [9], [10], [11], [12]. The current consensus for this observed QD intermittency is that non-charged QDs are fluorescent while charged QDs are not [7], [13]. The observed intermittency has been shown to be partially inhibited by small reducing agents such as β-mercaptoethanol (BME), dithiothreitol (DTT) and mercaptoethylamine (MEA) in mM concentrations [8], [14], [15]. Decreased blinking has also been accomplished by growth of thick semiconductor shells around the QD cores [16], [17]. Non-blinking CdZnSe/ZnSe QDs have recently also been reported, however, these QDs have a very broad emission spectra with three characteristic emission peaks making them non-useable for multiplexing applications [13].

In contrast to common perception, QDs have also been shown to photobleach under intense laser illumination [18], [19] and more recently even with Hg arc lamp illumination [20]. However, contrary to fluorescent dyes and proteins that display single step photobleaching from a fluorescent emitting state to a dark state, QDs have been shown to photobleach by a gradual blue-shifting of their emission color eventually reaching a permanent dark state [7], [18], [19]. This photobleaching of QDs is indicative of a process in which the QD core is gradually shrinking as a result of photooxidation of the core [7], [18], [19], [21], [22], a hypothesis which is supported by the reported slowing of blue shifting in a nitrogen atmosphere [19].

Our primary interest lies in applying QDs for single molecule multiplex labeling in biological applications. During the course of this work we also noticed that QDs will blue-shift under continuous illumination from even just a Hg arc lamp. In order to further understand the kinetics of this blue-shifting and to identify a simple biologically permissible cure for the blue-shifting, we set out to further study the extent of the QD emission color switching of biologically compatible streptavidin (sAv) conjugated QDs. In these studies we have combined the results from qualitative observations with a color CCD camera (CoolSNAP-Pro_cf_), by quantitative observations with an imaging spectrometer (ImSpector spectrograph) and finally at the single QD level by a combination of a Andor EMCCD and a QuadView image splitter designed for simultaneous multiplex imaging of QDs emitting at 565, 605, 655 and 705 nm. In all instances, these studies were done on QDs that had been non-specifically adsorbed to a glass coverslip and that were subject to continuous blue filtered (470 nm/40 nm wide bandpass excitation filter) light from a conventional 100 W Hg arc lamp and with use of a 150X, 1.45 NA microscope objective (Olympus). The light excitation power, at the back aperture of the objective, in this experimental set-up was 35 mW/cm^2^.

## Results

### Qualitative Direct Observations of QD Photobleaching at Bulk Concentrations

**Figure 1:**
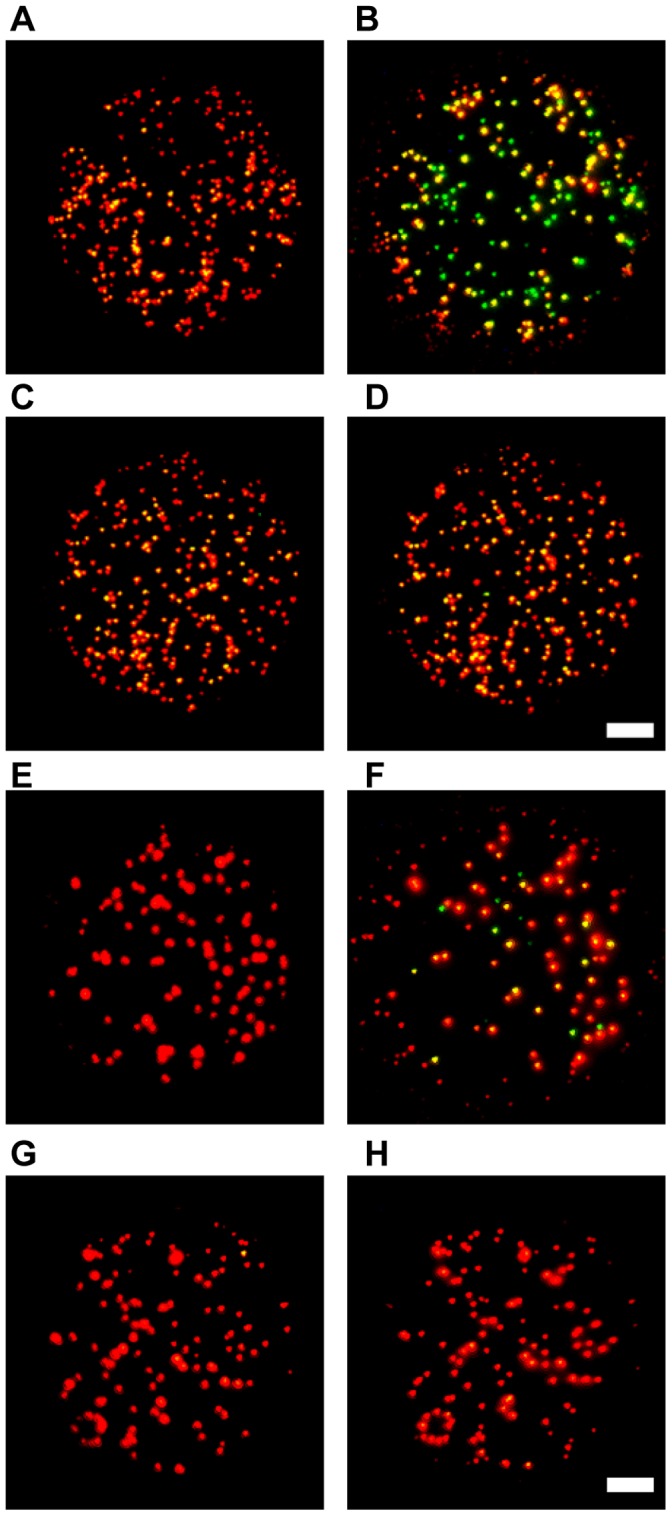
Fluorescence color switching of QDs emitting at 605 and 655 nm. QDs were non-specifically adsorbed to a glass coverslip and imaged under aqueous conditions on a CoolSNAP-Pro_cf_ color CD camera with 10 sec integration time. Left and right images were acquired about 7 minutes apart after continuous illumination with blue filtered light from a 100 W Hg arc lamp. QD605 (A) and QD 655 (C) in the absence of β-mercaptoethanol (BME), the QD emission color was observed to shift from an initial orange/red hue to a yellow-green hue. QD 605 (B) and QD655 (D) in the presence of 25 µM BME, the QD emission color was observed to be significantly stabilized to an orange/red hue for the duration of the experiment (space bar is 1 µm).

The photostability of biologically compatible QDs was first investigated at bulk densities (100s–1000s of adsorbed QDs in the field of view) by use of a color CCD camera and with 10 second camera integration times (Figures 1A and C). Under these detection settings, it was observed that QDs that are subject to continuous blue filtered excitation from a 100 W Hg arc lamp undergo a spectral blue switching over periods of minutes. The spectral shifting of QDs at the bulk level was furthermore observed to be significantly reduced in the presence of 25 µM BME (Figures 1B and D). Similar results were obtained with QDs emitting at 605 nm (Figures 1A and B) and 655 nm (Figures 1C and D).

### Quantitative Observations of QD Photobleaching at Bulk Concentrations

**Figure 2:**
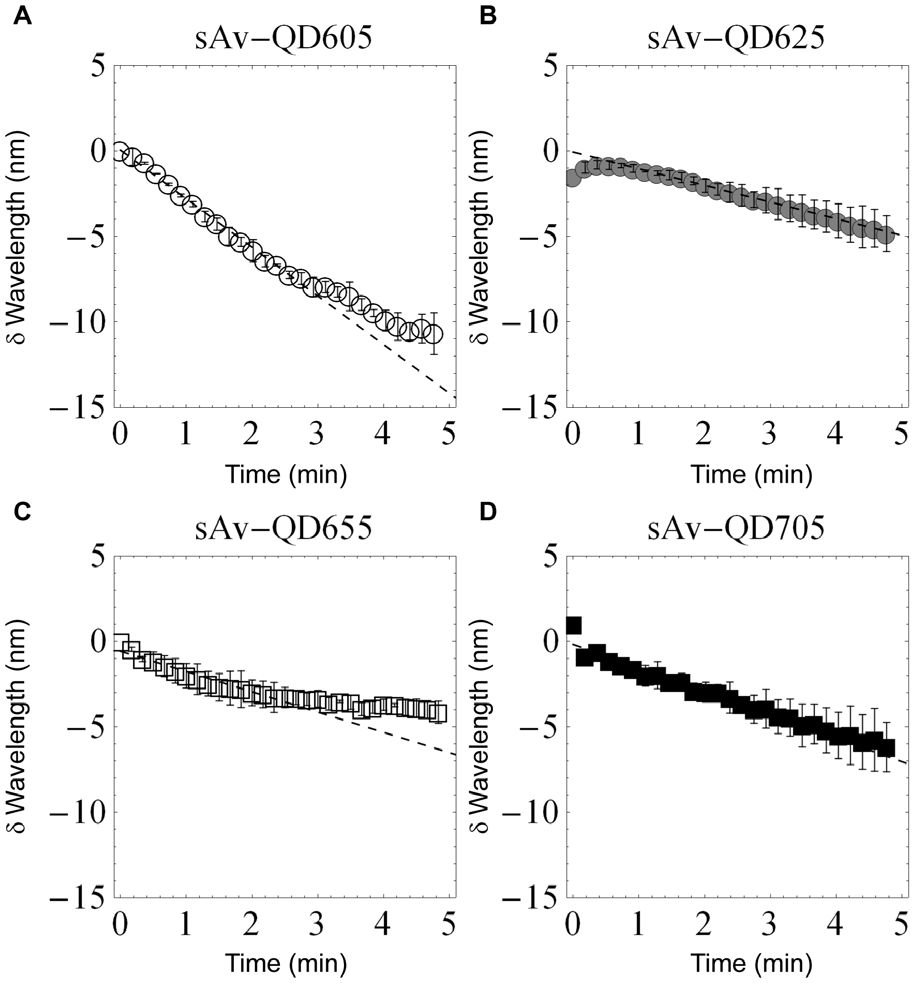
Spectral shifting of QDs measured by image spectrometer. The rate of spectral shifting in the absence of BME was investigated for QD605 (A), QD625 (B), QD655 (C), and QD705 (D). The reported data is the average of two experiments. The rates of spectral shifting in the absence of BME for the first 3 minutes were similar for all QDs. These rates were for QD605: −2.8 nm/min, QD625: −1.0 nm/min, QD655: −1.2 nm/min, and QD705: −1.4 nm/min.

**Figure 3:**
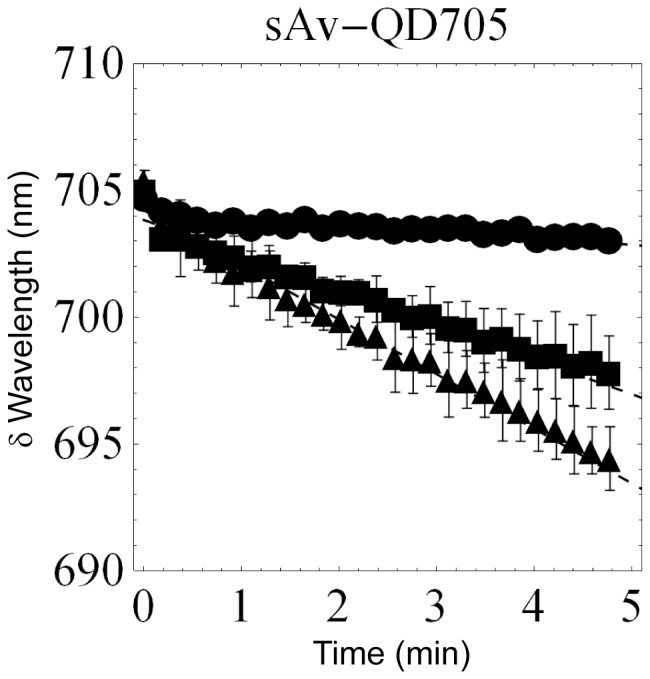
Fluorescence color switching of QDs observed with an ImSpector spectrograph. Fitted spectral peak positions of the fluorescence signal of QDs emitting at 705 nm as a function of time using the same illumination conditions as in Figure 1. The results shown are the average of two separate experiments in the absence of (squares), in the presence of (circles), and immediately following wash-out of (triangles) 25 µM BME. From this data, we determined the average spectral shifting rate of QDs emitting at 705 nm to be −1.3 nm/min in the absence of BME, −0.2±0.0 nm/min in the presence of 25 µM BME, and −2 nm/min after washout of BME.

In order to also obtain quantitative kinetic information on the observed color shifting at the QD bulk level, an image spectrometer in combination with an Andor EMCCD was used. In these experiments the spectral switching was evident by the continuous migration of the position of the fluorescence signal on the camera along the axis in the direction of the spectrometer response as a function of time. In order to be able to quantify the average rate of spectral shifting of QDs, the spectrometer response was calibrated by a combination of imaging samples with known emission wavelength (QDs with peak emission at 525, 585, 605, 625, 655, and 705 nm) and by exposing the spectrometer to respectively, laser illumination (473 nm) and illumination from a CoolLED (465 nm). The results of this calibration was a linear response (R^2^ = 0.998) over the visible spectrum and with a wavelength response of 1.63±0.03 nm/camera pixel (Figures S1 and S2). Using this calibration, we observed that the average spectral shifting rate of QD605s, QD625s, QD655s, and QD705s follow similar kinetics (Figure 2) with an approximately linear shifting rate over the first 3 minutes that was for QD605: −2.8 nm/min, QD625: −1.0 nm/min, QD655: −1.2 nm/min, and QD705: −1.4 nm/min. There are however differences as the rate of the blue-shifting of QD605 and QD655s decreases after about 3 minutes while the blue-shifting of QD625 and QD705s remains constant for at least the first 5 minutes. Furthermore, in the case of QD625s, we also observed a slight red-shifting in the emission over the first 30 seconds of illumination. Using this set-up we also investigated the photostabilizing effect of the small reducing agent BME on the emission of QD705 and found that the spectral rate decreased in the presence of 25 µM BME to −0.2 nm/min but that the spectral shifting rate again increased following wash-out of BME to −2 nm/min (Figure 3). In the presence of BME, the fluorescence intensity was also observed to increase as a function of time corresponding to a photoenhancement of the emission in the presence of continuous illumination (Video S1).

### Quantitative Observations of QD705 Photobleaching at Single QD Sensitivity

**Figure 4:**
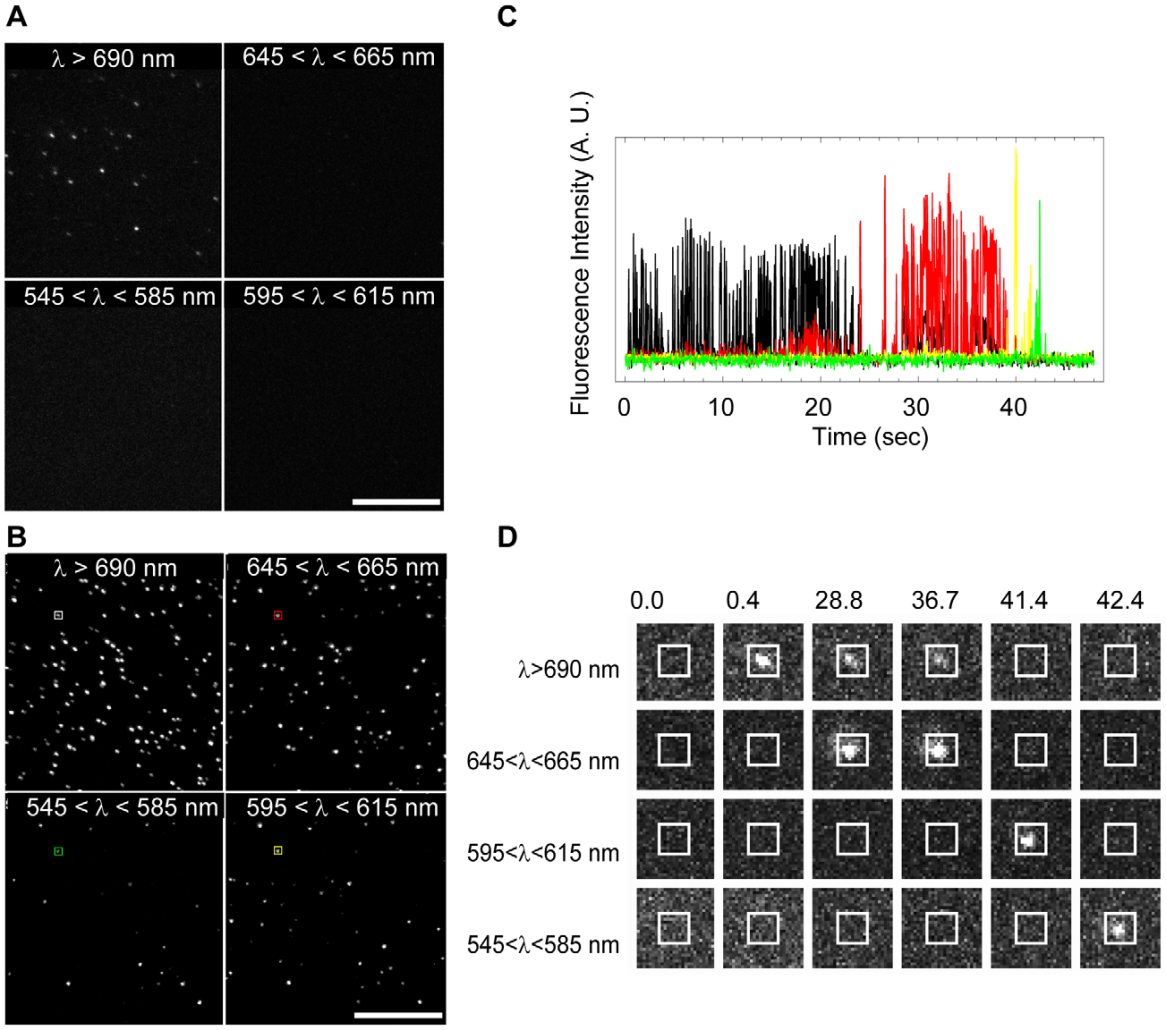
Fluorescence color switching of single QDs. (A) First image frame from a 1200 image frame sequence of QDs emitting at 705 nm and that had been non-specifically adsorbed to a glass coverslip. This image sequence was acquired with the QuadView image splitter allowing for simultaneous monitoring of 4 separate color channels, λ>690 nm, 645<λ<665 nm, 595<λ <615 nm, and 545<λ<585 nm. This image example was acquired in the absence of BME. (B) Maximum intensity projection of the same image sequence showing the total number of detected QDs in each of the four channels during the entire time course (here 48 seconds) of the experiment. (C) Spectral emission characteristics of selected highlighted single QD in bottom left image. Shown is the background subtracted integrated fluorescence signal contained within the highlighted region-of-interest (7×7 pixels) in the four separate color channels (λ>690 nm (black), 645<λ<665 nm (red), 595<λ<615 nm (yellow), and 545<λ<585 nm (green)) as a function of time. (D) Selected cropped snap shots of highlighted single QD showing the gradual color switching of a single QD across the entire available spectral range of the experiment.

**Figure 5:**
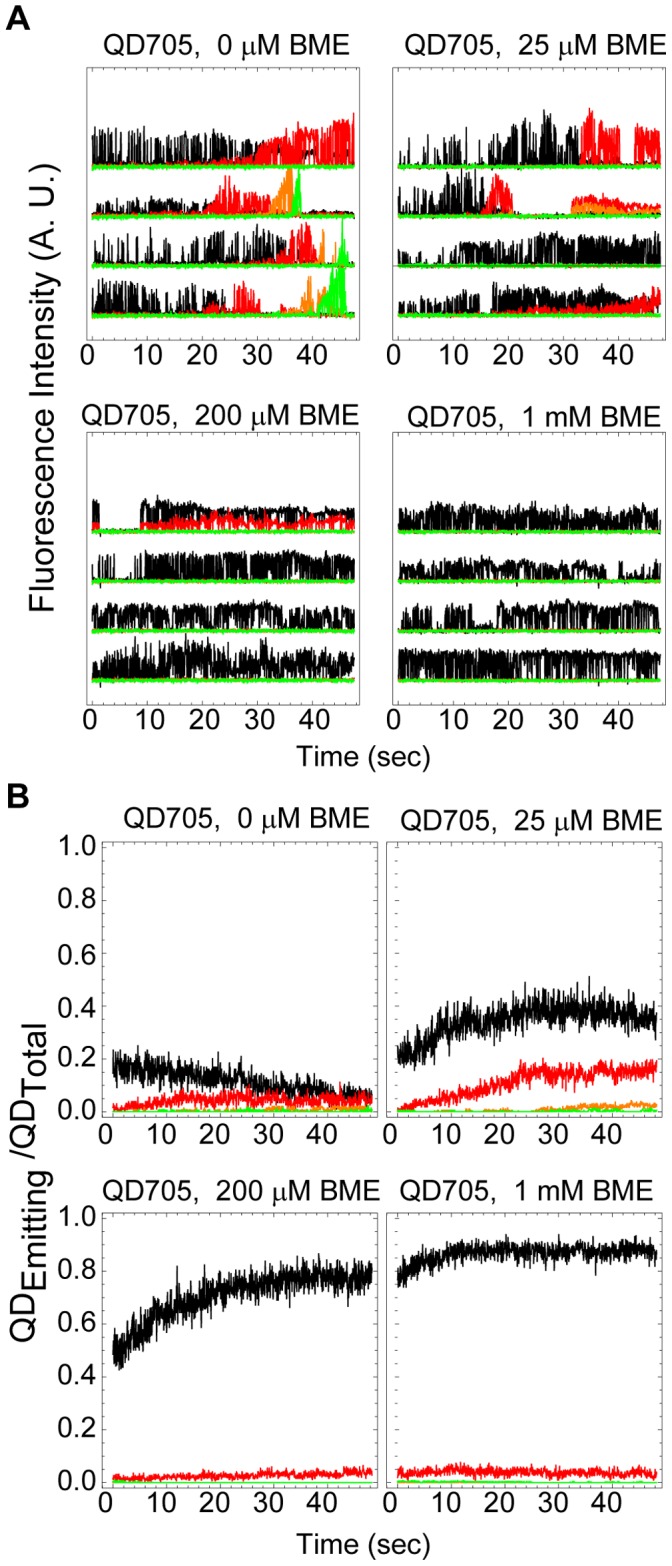
Quantification of single QD color switching. (A) Representative examples of the observed heterogeneity of the spectral color switching of single QDs as a function of time and of BME concentrations. Shown is the background subtracted integrated fluorescence signal of single QDs in the four separate color channels (λ>690 nm (black), 645<λ<665 nm (red), 595<λ<615 nm (yellow), and 545<λ<585 nm (green)) as a function of time.(B) Quantitative comparison of spectral emission characteristics of single QDs in an entire field of view as a function of time and of BME concentrations. Shown is the fraction of single QDs that are in their fluorescent emitting state relative to the total number of QDs that were ever observed in the upper spectral window (λ>690 nm)during the entire time course of the experiment (48 seconds), QD_Emitting_/QD_Total_, as a function of time and for each spectral color channel as above.

Having thus identified a potential biologically permissible cure for the blue-shfting problem we next proceeded to investigate the spectral shifting at single QD densities (10s–100s of adsorbed QDs in the field of view) for QDs emitting at 705 nm by use of a QuadView image splitter, containing fluorescence dichroics and bandpass filters to enable multi-species simultaneous imaging of single QDs emitting at 565, 605, 655, and 705 nm, in combination with an Andor EMCCD camera and with fluorescence excitation as before. The spectral down-shifting in these experiments is apparent by the successive migration of the fluorescence emission from single QDs from an upper spectral window designed for imaging at wavelengths greater than 690 nm, via a spectral window designed for imaging at wavelengths from 645 to 665 nm, through a spectral window ranging from 595 to 615 nm, and to a spectral window ranging from 545 to 585 nm, before finally disappearing completely (Figure 4). In these experiments, we observed that several instances where the gradual disappearance in the emission from a single QD in an upper spectral window coincided with the gradual appearance in emission of a single QD in an adjacent but lower spectral window and in an equivalent physical location on the camera chip, hence confirming that we were primarily observing the continuous spectral blue-shifting of single QDs. In these experiments, we regularly observed that single QDs would migrate across the entire available spectral emission range during the selected observation period of 1200 image frames that were acquired at around 25 Hz. An example of the observed spectral blue-shifting of a single QD is shown in Figures 4B–D. In these experiments it was also apparent that there is a large heterogeneity in the onset time, ranging from a few seconds to in some cases more than a minute, before single QDs will start their spectral blue-shifting (Figure 5A).We furthermore observed that in the case of experiments with single QD detection sensitivity, the onset of color switching is delayed but is not inhibited in the presence of 25 µM BME (Figure 5).

From these experiments it is also apparent that the small reducing agent BME enhances the photoemissive properties of QDs by two distinct means: 1) by minimizing the photobleaching rate of QDs, and 2) by increasing the time when QDs are in their emitting fluorescent state. The improvement effect of BME upon the intermittency frequency in this case is evident from the observed increase of the relative fraction of QDs that are fluorescent. For example, at the start of illumination and in the absence of BME, less than 20% of the total number of the 705 nm QDs are emitting fluorescence within the expected fluorescence spectral range (>690 nm), and where the fraction of fluorescent QDs within this spectral window remaining constant for the first 10 seconds after which a steady decrease of fluorescent QDs emitting at >690 nm coincides with an increase in the number of QDs that emit in the spectral window ranging from 645–665 nm. In contrast, the fraction of fluorescent QDs in the expected spectral window (>690 nm) at the onset of the experiment is about 20% in the presence of 25 µM BME, 45% in the presence of 200 µM BME, and 75% in the presence of 1000 µM BME, respectively. This effect of BME on the QD intermittency is consistent with earlier results [8] while the effect of BME on minimizing the photobleaching of QDs is in contrast to previously published results which reported that BME does prevent blue shifting but not photobleaching of QDs emitting at 525 nm [20]. In agreement with published data [23], we also see a photoenhancement effect such that the fraction of fluorescent QDs increases with time reaching about 35% (25 µM BME), 75% (200 µM BME), and 85% (1000 µM BME), respectively. This photoenhancement, however, is not observed in the absence of BME as the rate of photoenhancement is offset by the rate of spectral photoswitching (Figure 5B). At the two highest concentrations of BME, the observed QD color switching is also completely abolished, at least for the duration of these experiments (∼48 seconds).

## Discussion

In this work, we report further on the photostability of commercially available biologically compatible cadmium-selenide (CdSe) and hybrid cadmium-selenide (CdSe)/cadmium-telluride (CdTe) QDs having reported peak emissions at 605, 625, 655, and 705 nm, respectively. We show that these QDs, when subject to continuous blue-filtered excitation from a conventional 100 W Hg arc lamp, undergo a continuous blue-switching of the emission wavelength with QDs eventually reaching a permanent dark, photobleached, state. We furthermore show that the observed color switching is a single QD phenomenon as we observe a very large heterogeneity in the on-set of spectral shifting among single QDs, but that the rate of shifting accelerates once the spectral shifting has begun. We also show that we can reduce spectral downshifting of QDs by addition of even low doses (25 µM) of BME but that concentrations greater than 200 µM BME are typically required in order to completely prevent the QD color switching, at least for periods up to about 50 seconds. In this data it is also apparent that the amount of BME that is required to prevent QD blinking exceeds that which is required to prevent QD blue-shifting. We hypothesize that this is because BME serves dual purposes, 1) by acting as a weakly bound stabilizing ligand at the core/shell interface such as to prevent the occurrence of the ionized “dark state” of the QDs, and 2) by serving as a scavenger in solution for reactive oxygen species that are generated by the illumination. Worth noting is, however, that even though the required minimal dose of BME that was used is very low, this amount still corresponds to a molar excess of about 10^6^ compared to the amount of QDs.

The observed photoswitching is especially detrimental for multiplexing experiments performed at the single QD level as we regularly observe that single QDs undergo spectral shifts of 50 nm or more even after only a few to tens of seconds of continuous illumination conditions that are typically required for, e.g. high-speed single particle tracking experiments [3], [24]. In this work, we have, however, demonstrated that a simple cure to the observed blue-shifting in the QD emission spectra is to perform the experiments in the presence of small reducing agents, such as BME, whenever possible both in order to prevent QD color switching but also to enhance the frequency of time that the QDs are in their fluorescence emitting state. This is possible even in the case of experiments in live cells as addition of BME at low concentrations is considered to be physiologically permissible; in fact human stem cells are generally grown in 100 µM BME [25] and 50 µM BME has been shown to promote proliferation of human osteoprogenitor cells [26]. In all cases where imaging in the presence of BME is not possible, extreme care should be taken to ensure that the results are not influenced by QD spectral color switching. Finally it is worth noting that while the observed blue-shifting is a huge disadvantage in for example single particle tracking, which preferably requires photostability for long observation periods, it has recently been shown that much like the QD blinking can be taken advantage of to accomplish super-resolution imaging [6], [27], [28] so can the QD blue-shifting [22].

## Materials and Methods

### Materials

QDs were purchased in the form of Qdot® Strepavidin Conjugate Sampler Kits from Invitrogen (Carlsbad, CA), QDs with peak emission at 525, 585, 605, 625, and 655 consist of a CdSe/ZnS core-shell, and QDs with peak emission at 705 nm consist of a CdSe/CdTe core-shell. These QDs are water stabilized with an amphiphillic co-polymer [29] and has according to the manufacturer’s instructions 5–10 sAv covalently conjugated to the amphiphillic layer (Invitrogen, Carlsbad, CA) The hydrodynamic radii (mean) and polydispersity (±1 standard deviation) of these QDs has been found to be in the range of 10–12±1−2 nm based on the QD emission color as determined by FCS measurements (Arnspang et al. Multicolor single particle tracking with quantum dots, Manuscript submitted). All chemicals were reagent grade from Sigma-Aldrich (Brøndby, Denmark) except where specifically noted.

### Sample Preparation

QDs were diluted immediately prior to use in 50 mM sodium borate, pH 8.5, containing 1% BSA (Sigma A-7906) at concentrations ranging from 1–10 nM. In cases of experiments in the presence of BME the dilutions further contained the relevant volume of a 1 M stock solution of BME that was kept frozen. Diluted QDs were non-specifically adsorbed to the surfaces of sample chambers, composed of 3″ × 1″ glass or fused silica slides, two strips of double adhesive tape and a (25 mm)^2^ #1 ½ glass coverslip for a few minutes. Samples were subsequently extensively washed to remove non-adsorbed QDs with at least 4 sample volumes of 50 mM sodium borate, pH 8.5, containing 1% BSA and with and without the respective amount of BME.

### Microscopy

All samples were imaged on an Olympus IX-81 inverted microscope and with a 150X 1.45 NA UApo TIRFM Olympus microscope objective. This microscope was equipped for fluorescence excitation with both a 100 W Hg arc lamp and a pE-1 LED light source containing a high powered 465 nm LED array (CoolLED, Andover, UK). The excitation power at the objective back aperture plane and with a filter cube consisting of a HQ470/40 nm fluorescence excitation filter, a Q495LP dichromatic mirror (and a 500LP emission filter (Chroma Technology, Rockingham, VT) was measured at the back aperture of the objective to be about 35 mW/cm^2^ for the Hg lamp. Color fluorescence images were acquired with a CoolSNAP-Pro_cf_ color CD and using 20 second integration times. All other data was acquired on an Andor DV887-ECS/BV EMCCD (Andor, Belfast, UK) and using Andor IQ software. Single QD measurements were acquired using a QuadView image splitter with dichromatic mirrors at Q585LP, Q630LP and Q690LP nm and with D565/40 m nm, D605/20 m nm, and D655/20 m nm fluorescence emission bandpass filters from Chroma Technology (Rockingham, VT).

### Spectroscopy

Spectral measurements were acquired using a ImSpector spectrograph, Model V8/E (Andor, Belfast, UK) having resolution of 6 nm using a 30 µm slit. This spectrometer was coupled to the microscope through an optical fiber bundle of which we coupled the fluorescent emission from the microscope into one of the fibers and blocked the rest, imaging the output of the diffracted light through the spectrograph on the EMCCD. The fraction of fluorescent intensity coupled to the fiber was sufficient to track the spectral evolution of the integrated signal, which is essentially the horizontal pixel shift on the image plane. In order to analyze the migration of the peak position, the raw image data was converted to text format and imported into Mathematica for numerical analysis. In this analysis, we first averaged the fluorescence intensity along the part of y-axis that had a fluorescence intensity above the background intensity for all values of the x-axis. This data was then fit to a normalized 1D Gaussian, A+B/(2πσ^2^) Exp[−(x−x_0_)^2^/2σ^2^, where x_0_ is the mean peak position and σ is the standard deviation in the determination of the peak position.

### Image Analysis

The maximum intensity projection shows the brightest pixels in an entire image sequence and can be used to determine the total number of QDs that were ever in their fluorescent on state during the duration of the entire image sequence where the number of QDs is determined by use of a Particle Detector routine in ImageJ [30]. From this analysis we determined the fraction of QDs that are in their fluorescent state at time t, QD_Emitting_, relative to the total number of QDs that were ever on in the upper spectral window, λ>690 nm,during the entire image sequence, QD_Total_(λ>690 nm) where this fraction is defined as QD_Emitting_/QD_Total_(λ>690 nm).

### Supporting Information Available

Data analysis example of image spectrometer response and (Figure S1). Image spectrometer calibration data and results of image spectrometer response (Figure S2). Image and data analysis example of spectral color switching of single QD705 in absence of BME (Figure S3). Direct visual observation of the spectral color switching of QD705s with an image spectrometer (Video S1). Single QD705 color switching observed with a QuadView image splitter in the absence of BME (Video S2). Single QD705 color switching observed with a QuadView image splitter in the presence of 25 µM BME (Video S3).

## Supporting Information

Figure S1 Data analysis example of image spectrometer data. In this data, the direction of the image spectrometer response is along the x-axis. (top) Raw image data of QDs emitting at 705 at t = 0 and t = 1200 seconds in the absence of BME showing a slight left shift of the peak position following 1200 seconds of blue filtered illumination with a 100 W Hg arc lamp. (bottom) In order to analyze the migration of the peak position, the raw image data was converted to text format and imported into Mathematica for numerical analysis. In this example the observed average spectral shift corresponds to a blue-shift of 16±57 nm over the duration of the measurement.
(TIF)

Figure S2 Calibration data of image spectrometer. (top)Raw data of different QDs and light sources.(bottom) Plot of dependence of the known wavelength of the various standards on the change in pixel position (determined by 1D Gaussian fit to raw data in direction of the image spectrometer response where error bars are the Gaussian width from the fit. The results of this calibration was a linear response (R^2^ = 0.998) over the visible spectrum and with a wavelength response of 1.63±0.03 nm/camera pixel.
(TIF)

Figure S3 Image and data analysis example of spectral color switching of single QD705 in absence of BME. (top left) Maximum intensity projection of 1200 frame image sequence acquired at 24.84 Hz (total duration of 48.27 sec) of non-specifically adsorbed QD705 in each separate color channel. The maximum intensity projection shows the brightest pixels in an entire image sequence and can be used to determine the total number of QDs that were ever in their fluorescent on state during the duration of the entire image sequence where the number of QDs is determined by use of a Particle Detector routine in ImageJ. In the given example the total number of QDs detected in each spectral window were QD_Total_(λ>690 nm) = 122, QD_Total_ (645>λ>665 nm) = 77, QD_Total_ (595>λ>615 nm) = 47 and, QD_Total_ (545>λ>585 nm) = 23. (top right, bottom right and left) Images acquired at t = 0.04, 10.06, and 20.11 seconds, respectively, also showing the number of detected QDs, QD_Emitting_, in each spectral color window. From this analysis we can determine the fraction of QDs that are in their fluorescent state at time t, QD_Emitting_, relative to the total number of QDs that were ever on in the upper spectral window, λ>690 nm,during the entire image sequence, QD_Total_(λ>690 nm) where this fraction is defined as QD_Emitting_/QD_Total_(λ>690 nm) (see results in Figure 4b).
(TIF)

Video S1 Direct visual observation of the spectral color switching of QD705s with an image spectrometer. Time-lapse image sequences of spectral blue shifting of QD705s were observed with 200 msec integration at 1 Hz for 1200 images. The spectral blue-shifting is greater in the absence of BME (left) than in the presence of 25 µM BME (right). This is evident by the greater observed migration in the position of the fluorescence emission in the direction of the spectrometer response along the y-axis. (Left) In the absence of BME, the fluorescence emission is observed to migrate upwards as a function of time corresponding to a spectral blue-shifting of the emission. (Right) In the presence of 25 µM BME, the fluorescence emission is observed to migrate slightly upwards while the peak intensity is also observed to increase as a function of time corresponding to both a slight spectral blue-shifting and photoenhancement of the emission.
(AVI)

Video S2 Single QD705 color switching observed with a QuadView image splitter in the absence of BME. The time-lapse image sequence of QD705s was acquired with 10 ms integration at about 25 Hz for 1200 images in the absence of BME (scale bar = 10 µm; time stamp in seconds). At the onset of the movie, only QDs emitting in the upper spectral window (top left, >690 nm) are visible. Within a few seconds, however, single QDs become visible in the subsequent spectral window (top right, 645< × <665 nm) and after 10–15 seconds also in the following spectral window (bottom right, 595< × <615 nm), and finally in the last spectral window (bottom left, 545< × <585 nm) after 20–25 seconds before disappearing completely. Also clearly visible in the movie is the typical intermittency in the emission of single QDs; a process that also remains for QDs that have undergone spectral blue-shifting. The quantitative results of the spectral blue-shifting are found in Figure 4.
(AVI)

Video S3 Single QD705 color switching observed with a QuadView image splitter in the presence of 25 µM BME. The time-lapse image sequence of QD705s was acquired with 10 ms integration at about 25 Hz for 1200 images in the presence of 25 µM BME (scale bar = 10 µm; time stamp in seconds). At the onset of the movie, most QDs are emitting in the upper spectral window (top left, λ>690 nm). Within a few seconds, however, single QDs become visible in the subsequent spectral window (top right, 645< λ <665 nm) and after 25–30 seconds also in the following spectral window (bottom right, 595< λ <615 nm), and finally in the last spectral window (bottom left, 545< λ <585 nm) at 40–45 seconds before disappearing completely. Also clearly visible in the movie is the typical intermittency in the emission of single QDs; a process that also remains for QDs that have undergone spectral blue-shifting. The quantitative results of the spectral blue-shifting are found in Figure 4.
(AVI)
