# Correction: A Single Molecule Investigation of the Photostability of Quantum Dots

**DOI:** 10.1371/journal.pone.0124852

**Published:** 2015-04-09

**Authors:** The PLOS ONE Staff

The first author’s name is incorrect. The correct name is: Eva C. Anrspang. The correct citation is: Arnspang EC, Kulatunga P, Lagerholm BC (2012) A Single Molecule Investigation of the Photostability of Quantum Dots. PLoS ONE 7(8): e44355. doi: 10.1371/journal.pone.0044355
