# Correction: Correction: A Single Molecule Investigation of the Photostability of Quantum Dots

**DOI:** 10.1371/journal.pone.0138639

**Published:** 2015-09-18

**Authors:** The PLOS ONE Staff

The first author’s name in the text of the Correction notice published on April 9^th^, 2015 is misspelled. The publisher apologizes for the error. The correct name is: Eva C. Arnspang. The correct citation for the original article is: Arnspang EC, Kulatunga P, Lagerholm BC (2012) A Single Molecule Investigation of the Photostability of Quantum Dots. PLoS ONE 7(8): e44355. doi: 10.1371/journal.pone.0044355.
