# Supplementary figures and images for: A Single Molecule Investigation of the Photostability of Quantum Dots
Source: PLoS One. 2012 Aug 31;7(8):e44355. doi: 10.1371/journal.pone.0044355 (PMC3432116; doi:10.1371/journal.pone.0044355)

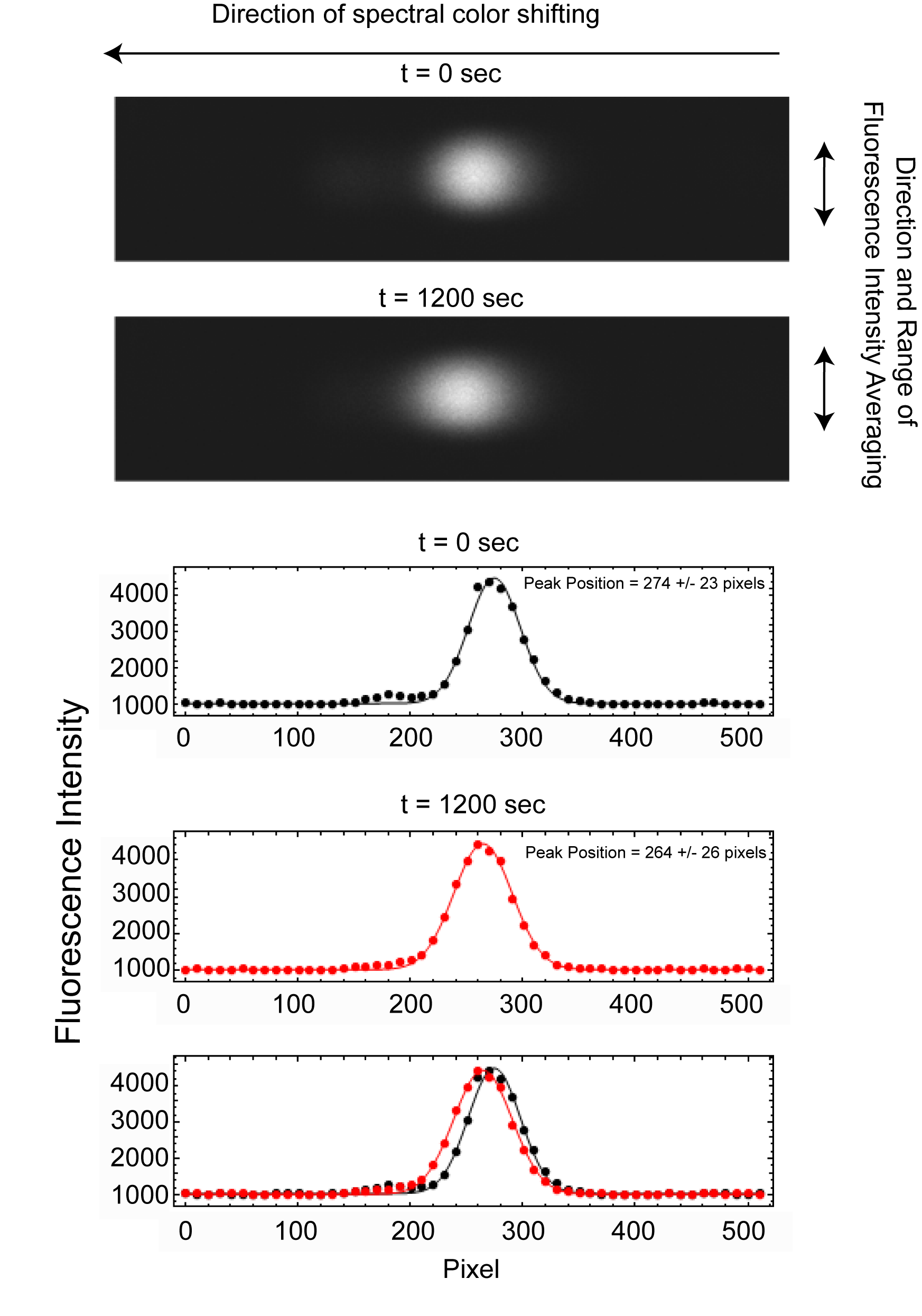

Supplement: Figure S1 — Data analysis example of image spectrometer data. In this data, the direction of the image spectrometer response is along the x-axis. (top) Raw image data of QDs emitting at 705 at t = 0 and t = 1200 seconds in the absence of BME showing a slight left shift of the peak position following 1200 seconds of blue filtered illumination with a 100 W Hg arc lamp. (bottom) In order to analyze the migration of the peak position, the raw image data was converted to text format and imported into Mathematica for numerical analysis. In this example the observed average spectral shift corresponds to a blue-shift of 16±57 nm over the duration of the measurement. (TIF) [file pone.0044355.s001.tif]

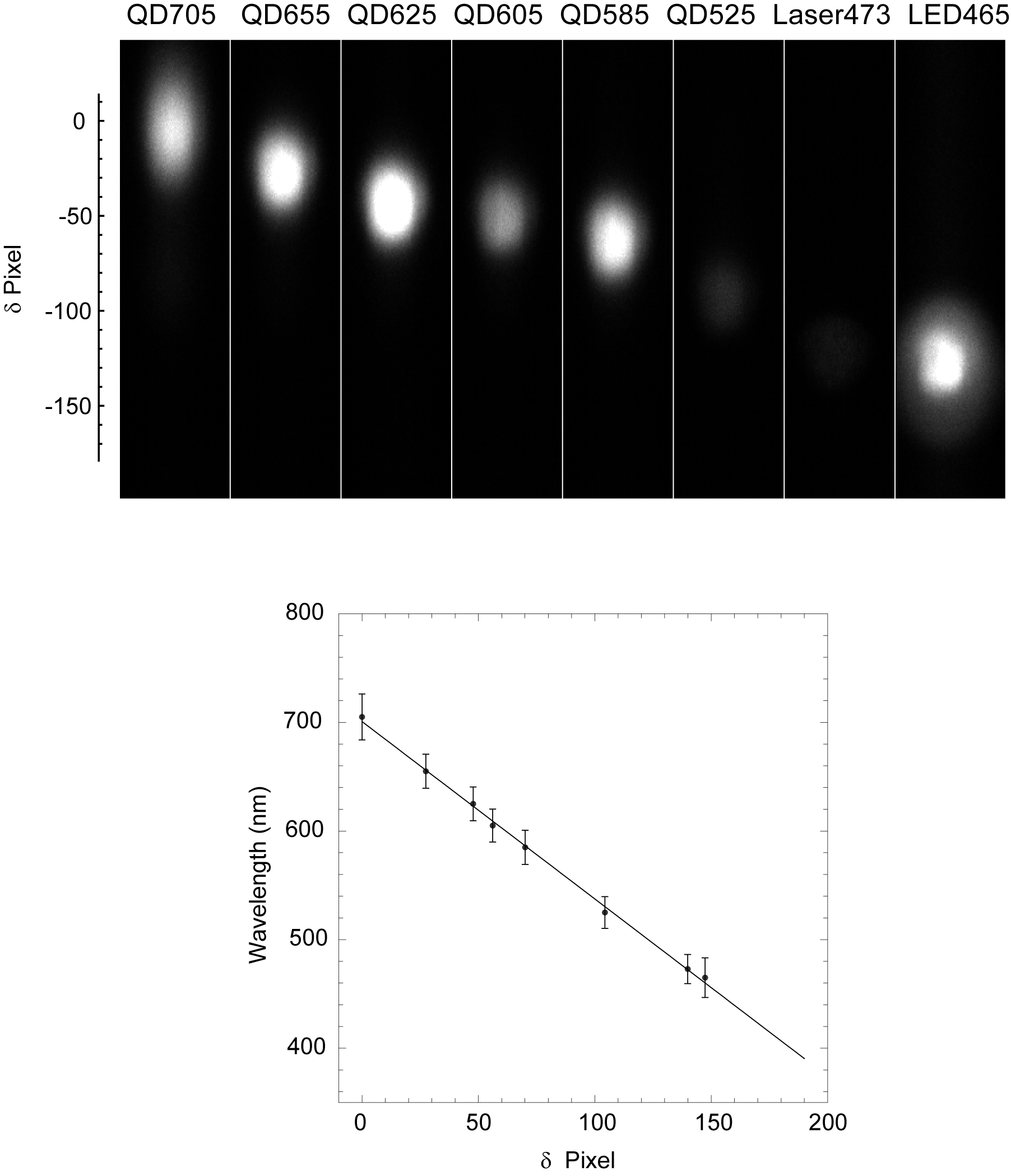

Supplement: Figure S2 — Calibration data of image spectrometer. (top)Raw data of different QDs and light sources.(bottom) Plot of dependence of the known wavelength of the various standards on the change in pixel position (determined by 1D Gaussian fit to raw data in direction of the image spectrometer response where error bars are the Gaussian width from the fit. The results of this calibration was a linear response (R2 = 0.998) over the visible spectrum and with a wavelength response of 1.63±0.03 nm/camera pixel. (TIF) [file pone.0044355.s002.tif]

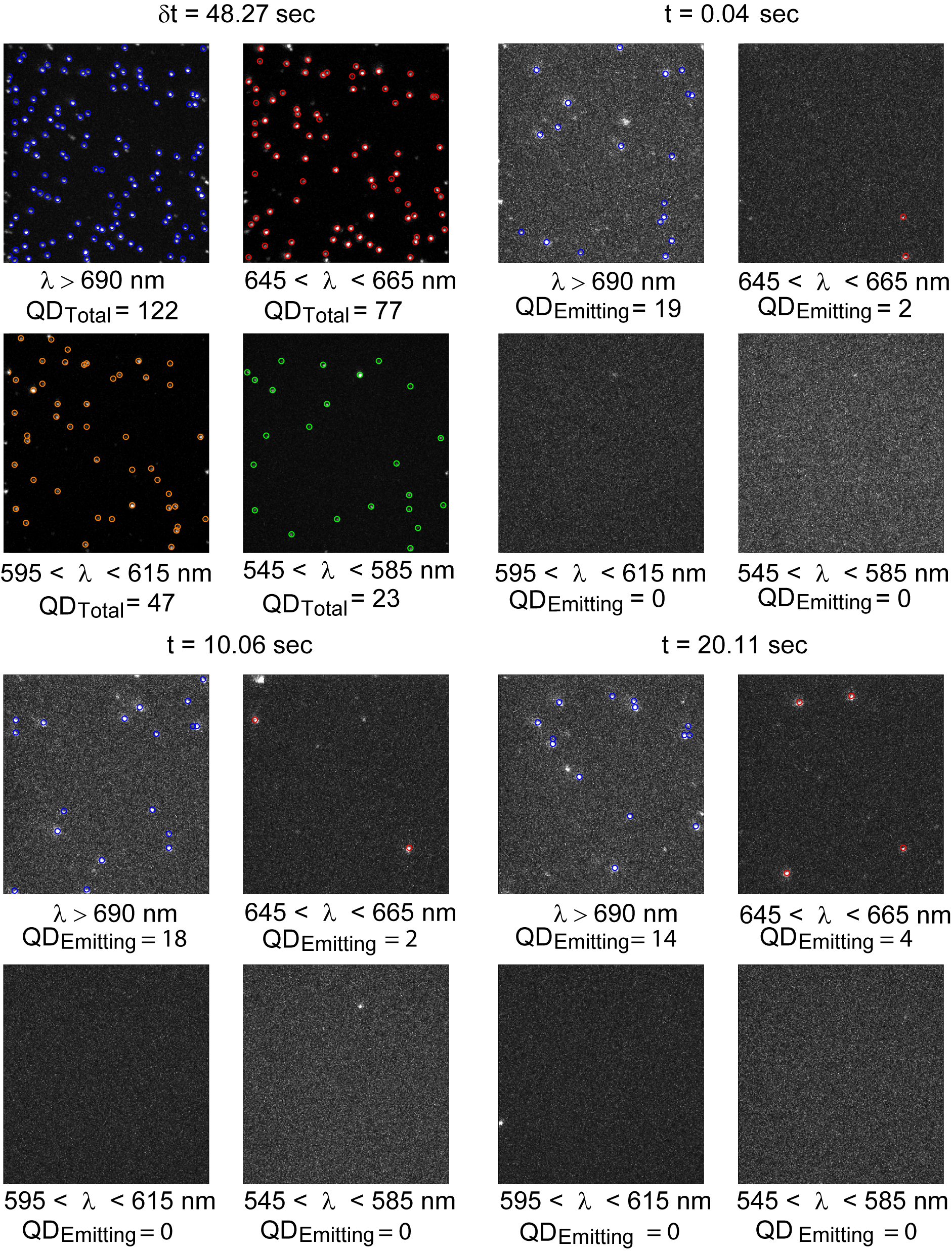

Supplement: Figure S3 — Image and data analysis example of spectral color switching of single QD705 in absence of BME. (top left) Maximum intensity projection of 1200 frame image sequence acquired at 24.84 Hz (total duration of 48.27 sec) of non-specifically adsorbed QD705 in each separate color channel. The maximum intensity projection shows the brightest pixels in an entire image sequence and can be used to determine the total number of QDs that were ever in their fluorescent on state during the duration of the entire image sequence where the number of QDs is determined by use of a Particle Detector routine in ImageJ. In the given example the total number of QDs detected in each spectral window were QDTotal(λ>690 nm) = 122, QDTotal (645>λ>665 nm) = 77, QDTotal (595>λ>615 nm) = 47 and, QDTotal (545>λ>585 nm) = 23. (top right, bottom right and left) Images acquired at t = 0.04, 10.06, and 20.11 seconds, respectively, also showing the number of detected QDs, QDEmitting, in each spectral color window. From this analysis we can determine the fraction of QDs that are in their fluorescent state at time t, QDEmitting, relative to the total number of QDs that were ever on in the upper spectral window, λ>690 nm,during the entire image sequence, QDTotal(λ>690 nm) where this fraction is defined as QDEmitting/QDTotal(λ>690 nm) (see results in Figure 4b). (TIF) [file pone.0044355.s003.tif]
